# Interpretable dynamic quantitative vascular morphometry features using SHAP for anti-angiogenic therapy response prediction

**DOI:** 10.1126/sciadv.aeb3543

**Published:** 2026-07-24

**Authors:** Kui Hu, Qian Cai, Jia Xu, Shuangquan Ai, Wuling Ou, Yulin Liu

**Affiliations:** ^1^ Department of Radiology, Hubei Cancer Hospital, Tongji Medical College, Huazhong University of Science and Technology, Wuhan, Hubei, China.; ^2^ Hubei Key Laboratory of Medical Information Analysis and Tumor Diagnosis & Treatment, Wuhan 430074, China.; ^3^ Thoracic Inner Department I, Hubei Cancer Hospital, Tongji Medical College, Huazhong University of Science and Technology, Wuhan, Hubei, China.; ^4^ College of Biomedical Engineering, South-Central Minzu University, Wuhan, Hubei, China.

## Abstract

Anti-angiogenic therapy benefits vary, with response rates of 40 to 70%, highlighting the need for early biomarkers to identify responders. We developed an automated machine learning framework that uses delta quantitative vascular morphometry features from standard contrast-enhanced CT to evaluate treatment response. This workflow combines automated tumor and vessel segmentation with feature extraction from routine scans for clinical use. Shapley additive explanations (SHAP)–based attributions identify key vascular and clinical features, providing meaningful, imaging-visible evidence aligned with therapy targets beyond traditional radiomics. Using baseline and follow-up CTs from 163 patients with lung cancer, we built three models using fivefold cross-validation, with the delta-merge model achieving high accuracy (area under the receiver operating characteristic curve = 0.842 internally, 0.806 externally). SHAP analysis uncovered an “arterial-dominant, venous-adaptive” pattern, where arterial involvement and venous recovery distinguish responders. This automated workflow and visualization support early, imaging-based response assessment and personalized treatment.

## Teaser

Interpretable delta quantitative vascular morphometry predicts response to anti-angiogenic therapy in lung cancer.

## INTRODUCTION

Anti-angiogenic therapy benefits vary with response rates of 40 to 70% (*1*), highlighting the need for early biomarkers to identify responders. Anti-angiogenic therapy induces a transient “normalization” of tumor vessels, which can enhance CD8^+^ T cell infiltration and improve delivery of cytotoxic agents into the tumor microenvironment (*2*); this provides a biological rationale for combining anti-angiogenic agents with chemotherapy and immunotherapy. This conceptual framework has been substantiated by the IMpower150 trial, which provided the first randomized evidence that incorporating atezolizumab into a bevacizumab-based chemotherapy backbone can further enhance clinical efficacy in the first-line treatment of advanced non–small cell lung cancer (*3*). Such combinations have become the standard of care in diverse real-world clinical settings (*4*, *5*), where anti-angiogenic agents serve as a foundational component to modulate the vascular microenvironment regardless of the concurrent cytotoxic or immune-modulatory regimen. Nevertheless, partial patients derive meaningful benefit, ranging from deep, durable remission to rapid resistance, underscoring the urgent need for predictive biomarkers to guide patient selection and avoid unnecessary toxicity (*6*). One important reason for the lack of robust biomarkers is the complexity of the tumor microenvironment, a dynamic ecosystem of cancer, stromal, and immune cells embedded in a disorganized, leaky, and tortuous vascular network (*7*, *8*); single-parameter biomarkers are unlikely to capture this multidimensional heterogeneity.

Radiomics, enabled by advances in digital imaging and computation, offers a noninvasive means to extract high-throughput, quantitative descriptors from routine medical images, providing broad spatial coverage, good standardization, and the potential for biological interpretability (*9*). While numerous computed tomography (CT), magnetic resonance imaging (MRI), and ultrasound studies have explored tumor-based radiomics or perfusion metrics for response prediction, comparatively few have focused directly on the vasculature—the primary target of anti-angiogenic therapy—although prior work has shown that quantitative vessel tortuosity and other vascular imaging biomarkers can distinguish benign from malignant lesions and predict immunotherapy response (*10*, *11*). The concept of delta radiomics, which analyzes feature changes between two time points, is now a well-established framework for capturing longitudinal treatment effects and often outperforms single–time point analysis (*12*, *13*). Building on these advances, we hypothesized that a framework specifically designed to capture dynamic vascular remodeling could outperform static baselines. However, prior vascular imaging studies have often relied on labor-intensive manual or semiautomated segmentation, limiting reproducibility and scalability for large clinical cohorts.

Furthermore, the “black-box” nature of many radiomics models hinders clinical trust. To address these gaps, we developed a fully automated, end-to-end pipeline that integrates: (i) delta quantitative vascular morphometry feature (QVMF): shifting the focus from static snapshots to dynamic treatment-induced changes; (ii) high-throughput automation (*14*–*17*): using deep learning for robust tumor and vessel segmentation, ensuring high reproducibility and reducing workload; and (iii) Shapley additive explanations (SHAP)–based interpretability (*18*, *19*): providing transparent, anatomically grounded explanations for model predictions. We applied this framework to a real-world cohort to develop an interpretable delta-merge model that predicts response to anti-angiogenic therapy–based regimens while balancing predictive performance, model complexity, and clinical usability.

## RESULTS

### Patient characteristics

**Fig. 1.**
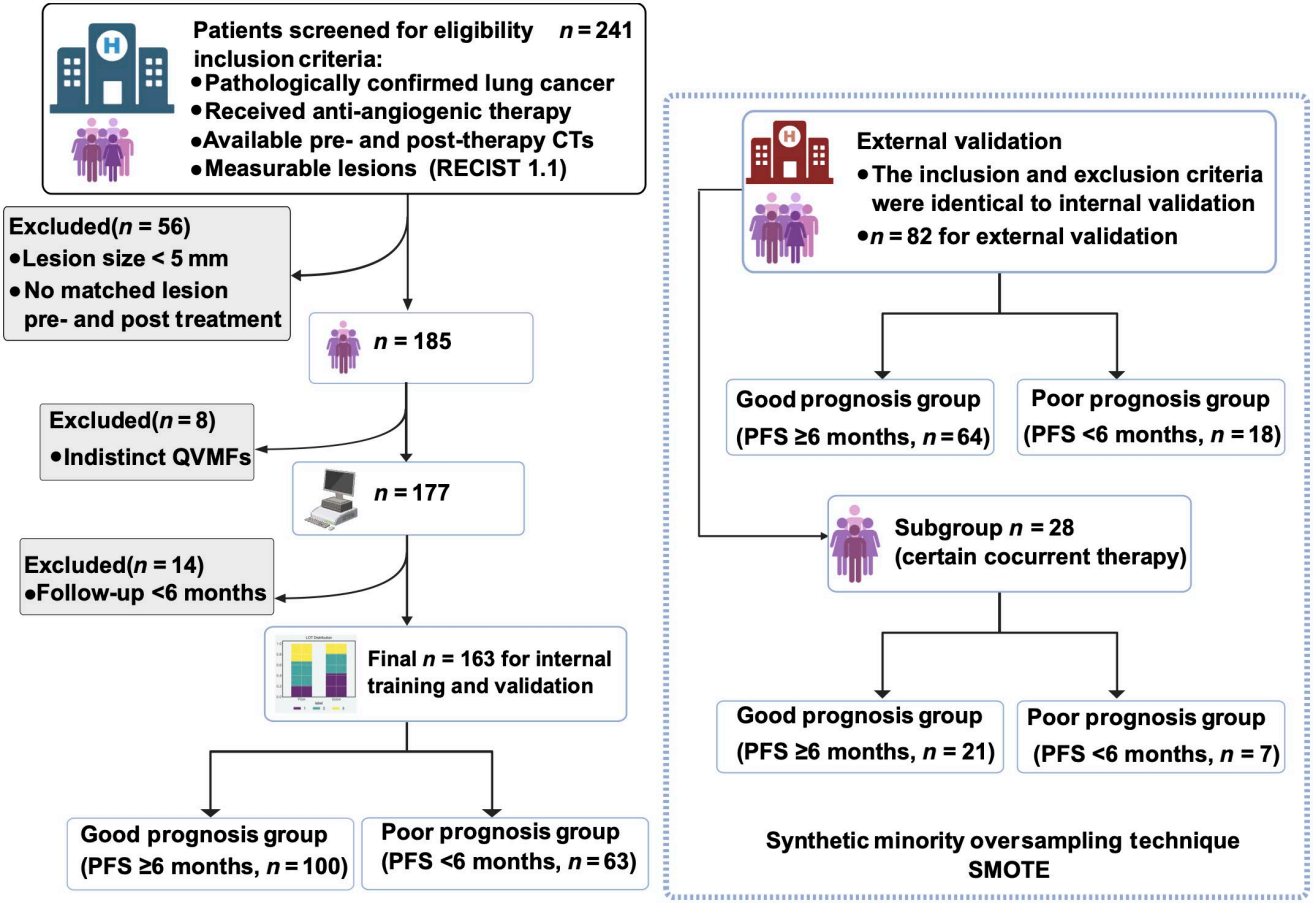
Patient enrollment flowchart. Patient selection process showing the internal training and validation cohort (*n* = 163) and external validation cohort (*n* = 82). A subgroup analysis was performed in 28 patients receiving concurrent therapy.

We enrolled a total of 245 patients in this study, comprising a primary training/internal validation cohort of 163 patients from Hubei Cancer Hospital and an independent external validation cohort of 82 patients from Wuhan Union Hospital (Fig. 1). Table 1 summarizes the detailed demographic and clinical characteristics of the primary cohort. The baseline characteristics of the external validation cohort were generally comparable to the primary cohort and are detailed in table S1. Given the sample size of the primary cohort, we used a fivefold cross-validation framework to develop a robust model, followed by validation on the external cohort using the synthetic minority oversampling technique (SMOTE) (*20*).

**Table 1.** Clinical characteristics of patients stratified by treatment response. Note: Lactate dehydrogenase, LDH (U/L); data are presented as means ± SD for continuous variables (age, BMI, and LDH) or number (%) for categorical variables. *P* values were calculated using Student’s *t* test or Mann-Whitney *U* test for continuous variables, the Chi-square test for nominal categorical variables (sex, smoking, histology, mutation, metastasis, and concurrent treatment), and the Mann-Whitney *U* test for ordinal variables (ECOG, LOT).

Characteristic	Poor response (*n* = 63)	Good response (*n* = 100)	*P* value
Age (years)	61.2 ± 9.9	61.5 ± 10.9	0.799
BMI (kg/m^2^)	22.3 ± 3.3	23.3 ± 3.1	0.061
Sex			0.852
Male	31 (49.2%)	52 (52.0%)	
Female	32 (50.8%)	48 (48.0%)	
ECOG			0.212
0	1 (1.6%)	3 (3.0%)	
1	44 (69.8%)	75 (75.0%)	
2	6 (9.5%)	7 (7.0%)	
Unknown	12 (19.1%)	15 (15.0%)	
Smoking history			0.472
Never	46 (73.0%)	74 (74.0%)	
Current/former	17 (27.0%)	26 (26.0%)	
Histology			0.109
Adenocarcinoma	50 (79.4%)	91 (91.0%)	
Squamous cell carcinoma	4 (6.3%)	3 (3.0%)	
Small cell carcinoma	7 (11.1%)	6 (6.0%)	
Others	2 (3.2%)	0 (0.0%)	
Genetic mutation			0.518
Wild type	16 (25.4%)	23 (23.0%)	
EGFR	38 (60.3%)	65 (65.0%)	
ALK	1 (1.6%)	1 (1.0%)	
ROS1	0 (0.0%)	3 (3.0%)	
KRAS	2 (3.2%)	4 (4.0%)	
Others	6 (9.5%)	4 (4.0%)	
Brain metastasis			0.682
Yes	21 (33.3%)	29 (29.0%)	
LDH (U/L)	243.2 ± 68.4	224.9 ± 56.8	0.159
Line of therapy			0.002
First-line	13 (20.6%)	44 (44.0%)	
Second-line	29 (46.0%)	37 (37.0%)	
Third-line or later	21 (33.3%)	19 (19.0%)	
Concurrent treatment			0.774
Chemotherapy	28 (44.4%)	42 (42.0%)	
TKI	19 (30.1%)	30 (30.0%)	
Chemo + immuno	10 (15.9%)	16 (16.0%)	
Monotherapy	2 (3.2%)	8 (8.0%)	
Immunotherapy	2 (3.2%)	3 (3.0%)	
Others	2 (3.2%)	1 (1.0%)	

As summarized in Table 1, detailed analysis of clinical characteristics showed no significant differences in age, sex, smoking history, histology, concurrent treatment regimens, or brain metastasis status between responders and nonresponders (*P* > 0.05). However, line of therapy (LOT) differed significantly (*P* = 0.002), with responders more likely to be in earlier treatment lines (44.0% first-line) compared to nonresponders (20.6% first-line). This finding identified LOT as a critical clinical covariate, motivating its inclusion in our multivariate models.

We assessed 241 patients for eligibility. Exclusion criteria included the following: inadequate CT image quality, lesions too small for AI delineation, progression-free survival (PFS) follow-up <6 months, and extensive pulmonary conditions affecting vascular segmentation (e.g., extensive bullae/emphysema, massive atelectasis, and bilateral pneumothorax), or pulmonary conditions affecting the tumor or peritumoral region (e.g., bullae/emphysema in tumor or peritumoral region, pneumothorax on tumor-bearing side, and extensive pneumonia/interstitial pneumonia affecting peritumoral region). We included 163 patients in the final analysis.

### External validation

In addition to the internal cohort, we conducted an external validation with 82 patients. This external dataset included patients treated with a combination of anti-angiogenic therapy, immunotherapy, Tyrosine kinase inhibitors (TKI), or chemotherapy. Among these, chemotherapy combined with immunotherapy forms a distinct subgroup for a separate model validation. Because of class imbalance in the external dataset, we used the SMOTE algorithm to balance the samples for the validation process. This external validation further corroborates the model’s generalizability across diverse clinical settings.

### Univariate and multivariate analysis

**Fig. 2.**
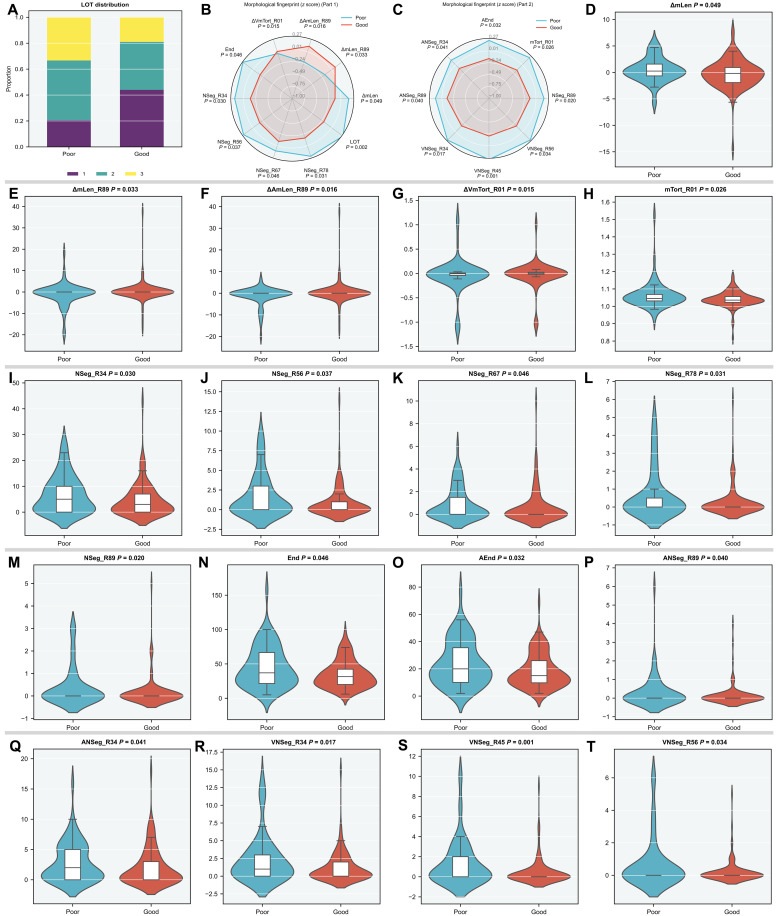
Univariate analysis of treatment line distribution and QVMFs between good (pink red) and poor (light blue) responders. (**A**) LOT distribution. Stacked bar chart showing patient proportions across LOT 1, 2, and 3. This panel summarizes baseline treatments, prior regimens across cohorts, and differences in treatment history between response groups. Radar charts show distinct vascular morphological patterns for the two outcome groups, providing visual profiles. (**B**) Features include: ΔAmLen_R89, ΔVmTort_R01, ΔmLen_R89, ΔmLen, LOT, NSeg_R78, NSeg_R67, NSeg_R56, NSeg_R34, and End. (**C**) Features are: mTort_R01, NSeg_R89, VNSeg_R56, VNSeg_R45, VNSeg_R34, ANSeg_R89, ANSeg_R34, and AEnd. (**D**) through (**T**) feature violin plots comparing poor and good response groups for significant morphological features. Each plot shows the QVMF’s name, the *P* value from the Mann-Whitney *U* test, and the data distribution via kernel density estimation, with embedded box plots for the median, interquartile range, and whiskers. These panels, linked to morphological fingerprints (B and C) and radar charts, offer detailed univariate insights into individual feature distributions. Features identified through univariate analysis (*P* < 0.05) were standardized using *z* scores, and mean values for each group were calculated. Abbreviations: Δ indicates delta features (post minus pre); m, mean; A, artery; V, vein; Len, length; Seg, segment; Tort, tortuosity; N, number; R01, radius bin 0-1.

To identify key prognostic factors, we first conducted univariate analysis to assess the association of each clinical and vascular morphometric feature with 6-month PFS. This initial screen identified 18 features that were significantly associated with the outcome (*P* < 0.05), including the clinical variable LOT and 13 baseline and four delta QVMFs, summarized in table S2. These analyses revealed significant differences in both baseline vascular architecture and dynamic posttreatment changes between response and nonresponse groups. We created a composite figure to visualize these partial key findings (Fig. 2).

We then included these significant features in a multivariate logistic regression analysis to evaluate their independent predictive value. The final multivariate model revealed that “LOT,” the baseline feature “mTort_R01,” and one delta feature “ΔmLen” remained significant independent prognostic factors (table S2).

### Paired *t* test analysis

**Fig. 3.**
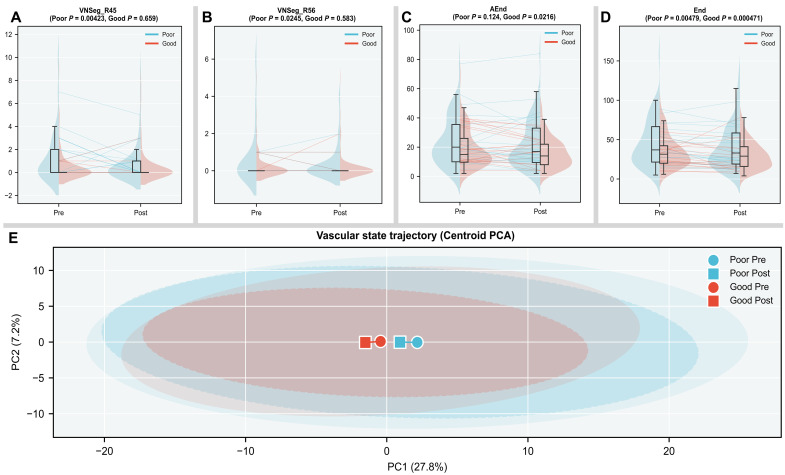
Longitudinal changes in vascular features and overall state dynamics in response to treatment. (**A** to **D**) Paired violin plots show changes (Δ) from pre- to posttreatment in key QVMFs. Distributions display median and interquartile range, with individual patient measurements connected by semitransparent lines. Features include VNSeg_R45, VNSeg_R56, AEnd, and End, highlighting response-specific patterns of vascular change in the poor- and good-response groups. (**E**) The distribution and trajectory of vascular states are visualized through principal component analysis (PCA) of standardized QVMFs. Shaded ellipses represent 95% confidence regions around group centroids for the poor (blue) and good (red) groups at pre- and posttreatment (circles and squares). Arrows link pre- and posttreatment centroids, indicating the direction and magnitude of shifts in vascular state (PC1: 27.8%; PC2: 7.2%).

Paired *t* test analysis of pre- and posttreatment QVMFs revealed distinct response patterns between groups within the short-term treatment window (table S3). Poor responders (light blue) showed significant reductions in vascular network density, particularly in venous segments at radius bins 4-5 and 5-6 (Fig. 3A, VNSeg_R45: *P* = 0.004; Fig. 3B, VNSeg_R56: *P* = 0.024) after 4 to 6 weeks of therapy initiation. Good responders (pink red) demonstrated selective end point reduction, primarily affecting arterial end points (Fig. 3C, AEnd: *P* = 0.022) while preserving network architecture during this early treatment period. Overall cohort analysis confirmed significant treatment effects on vascular end point reduction (Fig. 3D, End: *P* < 0.01), establishing quantitative evidence of rapid therapy–induced vascular remodeling occurring within the first follow-up assessment window.

Principal components analysis of QVMFs revealed distinct distributional patterns between responders and nonresponders, as indicated by the separation of 95% confidence regions around group centroids (Fig. 3E). Treatment effects were captured by centroid trajectories from baseline to follow-up, showing an overall shift toward vascular simplification in both groups, with more coherent changes observed in responders than in nonresponders.

**Fig. 4.**
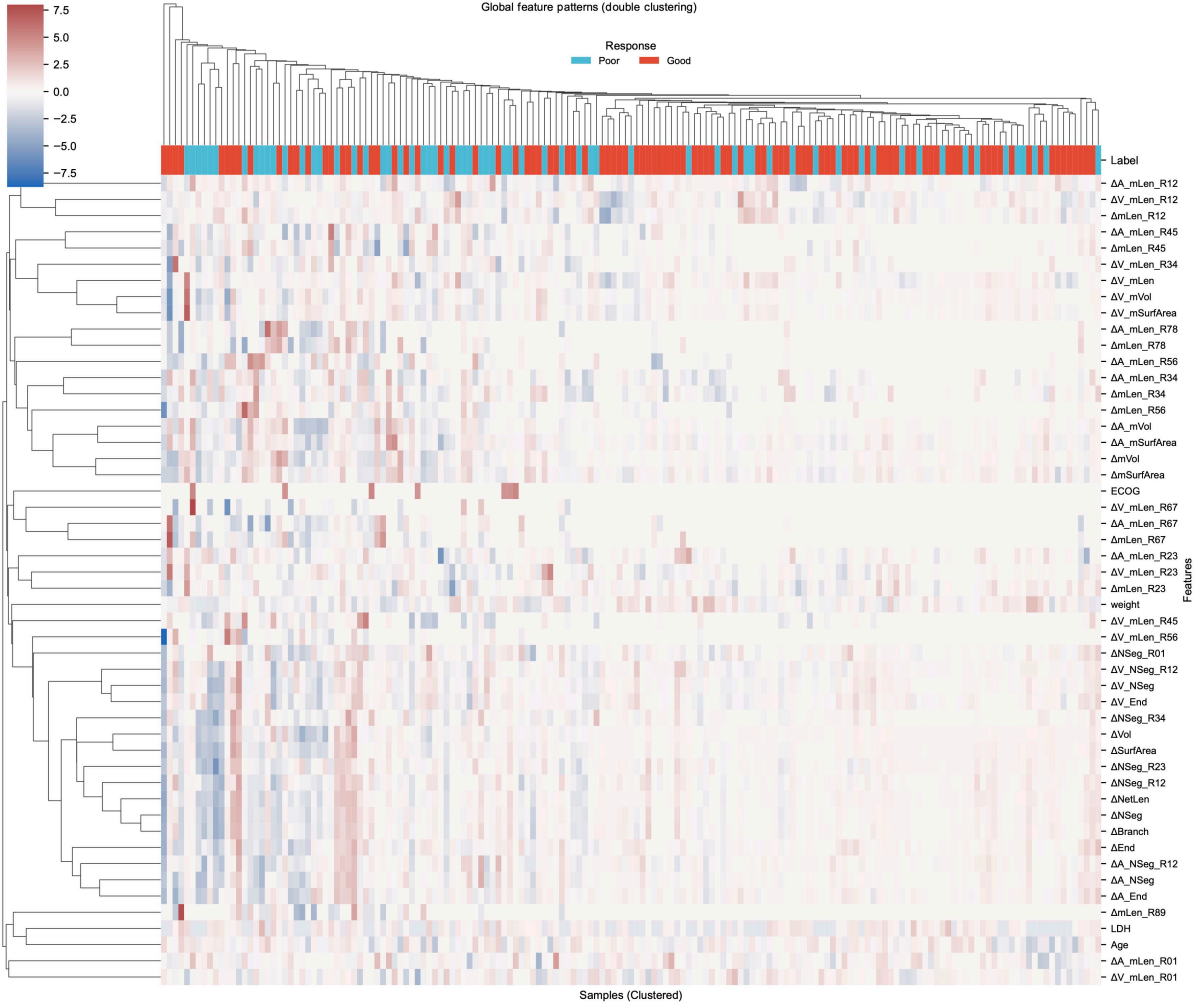
Global pattern clustering analysis of vascular morphological features. The hierarchical clustering heatmap shows overall patterns across all samples and features. All numeric features from the delta data were selected (if >50 features, the top 50 by variance), standardized to *z* scores, and used to create the heatmap. Double clustering was applied: sample clustering (columns) and feature clustering (rows), using hierarchical clustering with Euclidean distance. The “vlag”color scheme (blue-white-red), centered at 0, represents standardized means. The top color bar indicates sample groups (blue, poor response; red, good response). The dendrograms on the left and top sides display clustering of features and samples. This analysis identifies similar vascular patterns, coexpressed feature modules, and whether sample clusters align with treatment response.

Hierarchical clustering analysis of treatment-induced vascular remodeling revealed distinct patterns of feature expression across response groups (Fig. 4). Samples with similar delta feature profiles tended to cluster together, and feature clustering identified groups of covarying delta features, suggesting coordinated changes in vascular morphology following treatment.

### Feature selection and model development

**Fig. 5.**
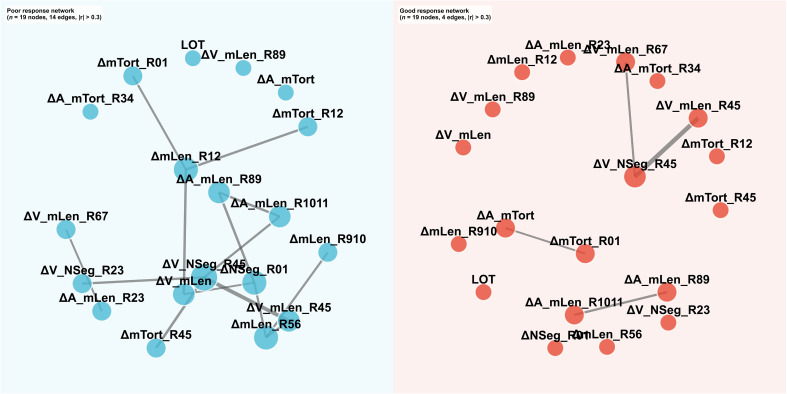
Key vascular morphological feature correlation network analysis. Feature correlation networks for the poor and good response groups were constructed separately using 19 key features selected by LASSO regression. Using Spearman correlation (robust to outliers), connections were made only if |r| > 0.3. Networks are undirected, weighted graphs: Nodes represent features, and edge thickness reflects the strength of the correlation. Node sizes vary with degree, highlighting core features with strong associations. Layout uses a force-directed algorithm with *k* = 3.0 and 200 iterations. The poor (left light blue) group appears denser, indicating stronger feature links; the good (right light red) group is sparser, indicating independence. Titles show nodes, edges, and threshold.

**Fig. 6.**
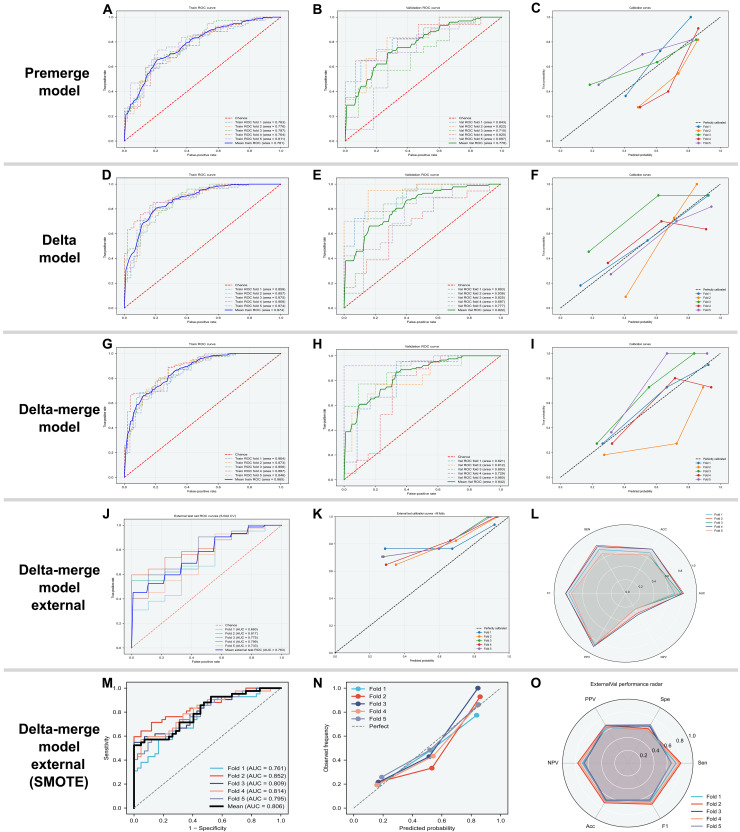
Comprehensive evaluation of three predictive models: Premerge, Delta, and Delta-merge using fivefold cross-validation. (**A**) to (**C**) display ROC and calibration curves for the premerge model (train AUC = 0.781, validation AUC = 0.778). (**D**) to (**F**) show these for the Delta model (train AUC = 0.874, validation AUC = 0.822). (**G**) to (**I**) present the same for the Delta-merge model (train AUC = 0.865, validation AUC = 0.842). (**J**) to (**L**) illustrate external validation of the Delta-merge, including ROC [(J) AUC = 0.763; (L) NPV = 0.297]. (**M**) to (**O**) illustrates external validation of the Delta-merge using SMOTE, including ROC [(M) AUC = 0.806; (O) NPV = 0.707].

Feature processing used normalization and least absolute shrinkage and selection operator (LASSO) regression for dimensionality reduction, selecting 10, 20, and 19 features for premerge, delta, and delta-merge models, respectively (LASSO regression plots for three models are shown in fig. S1). Correlation network analysis of the 19 LASSO-selected features from the delta-merge model revealed distinct network structures between response groups (Fig. 5). The poor response network exhibited more complex connectivity compared to the good response network, suggesting different patterns of feature covariation between groups. Selected features and their regression coefficients are detailed in table S4. Among the five machine learning algorithms tested (results shown in table S5), logistic regression achieved the best performance, with a validation area under the receiver operating characteristic (ROC) curve (AUC) of 0.842. The model hierarchy showed delta-merge (AUC = 0.842) > delta (AUC = 0.822) > premerge (AUC = 0.778), confirming the superiority of dynamic vascular features (Fig. 6). The average AUC of delta-merge on the external test is 0.806. ROC analysis demonstrated robust performance with minimal overfitting for the delta-merge model.

### External validation

To assess generalizability, we evaluated the models in an independent external cohort of 82 patients from Wuhan Union Hospital, whose CT scans differed from the training data in contrast-enhancement phase (arterial versus venous) and in slice thickness (approximately twofold thicker than in the training set). On this original, non-resampled external cohort, the premerge, delta, delta-merge, and delta-merge subgroup models achieved average AUCs of 0.661, 0.717, 0.763, and 0.799, respectively (fig. S2). These values were slightly lower than the internal validation AUCs because of differences in image acquisition and treatment but still showed good discriminative ability across varying conditions. The mean negative predictive value (NPV) of the delta-merge model stayed below 0.60, reflecting a few nonresponders and a skewed class distribution in the external cohort.

Because of class imbalance, a SMOTE-based analysis was conducted. Under this balanced scenario, models achieved average AUCs of 0.638, 0.763, 0.806, and 0.850, with ROC curves in fig. S3. The delta-merge model performed best, especially in the anti-angiogenic + chemotherapy + immunotherapy subgroup. Although AUCs were similar to the original cohort, the mean NPV for the delta-merge model increased to 0.707 (0.775 in the subset; table S6), indicating that low NPV in raw data is mainly due to class imbalance and few negatives, not a lack of discriminative signal.

Together, the original and SMOTE-based external analyses indicate that the delta-merge model retains good AUC performance across institutions and CT acquisition protocols. In contrast, the sensitivity analysis provides a more nuanced assessment of model robustness to class-imbalance effects than simple cross-study AUC comparisons alone.

### Model interpretation

#### SHAP analysis of feature importance

To ensure transparency and interpretability of our models, we used the SHAP framework. The following analysis focuses on the best-performing delta-merge model to illustrate the model’s decision-making process.

**Fig. 7.**
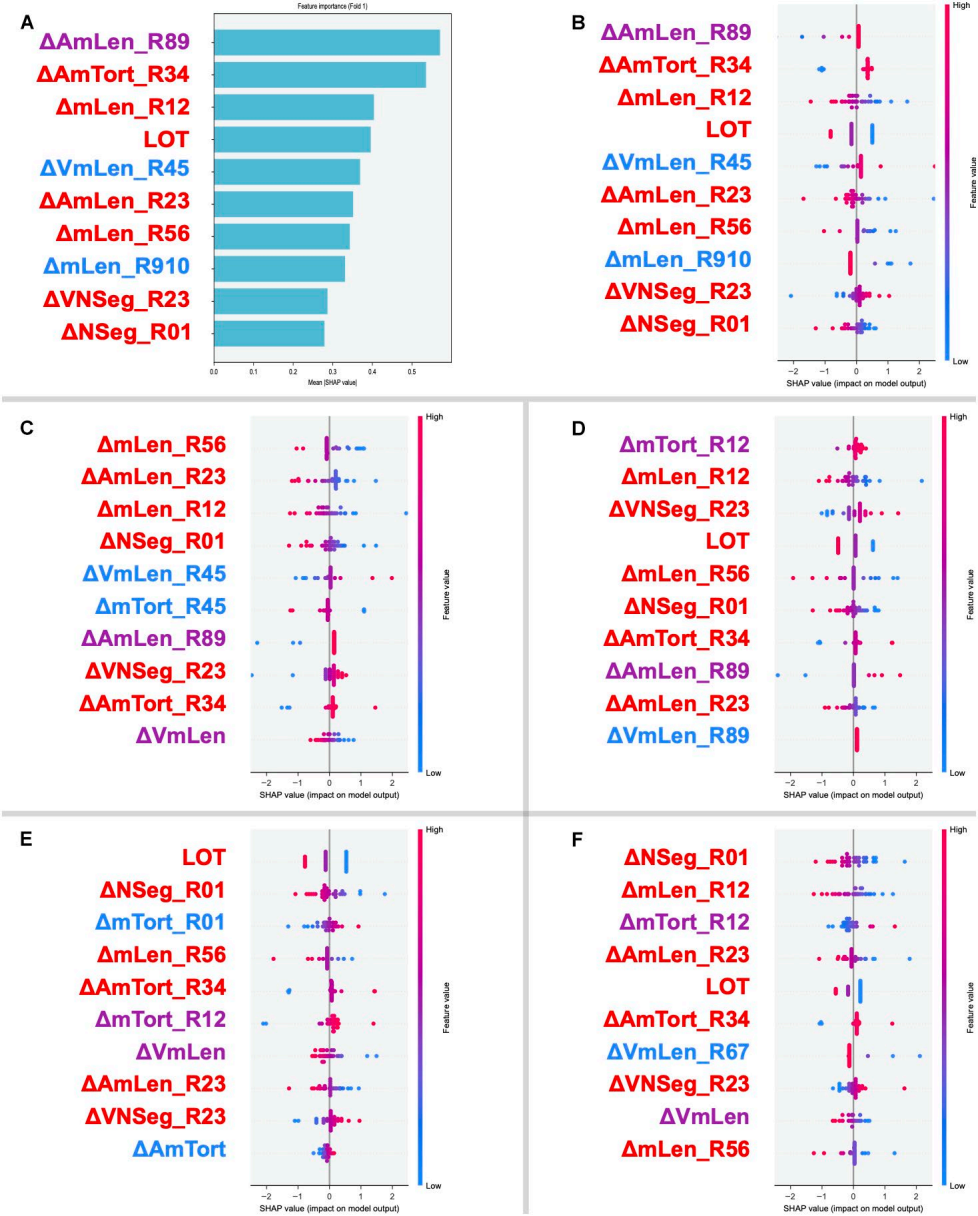
SHAP analysis of feature importance and consistency across folds. (**A** and **B**) Feature importance bar chart (A) and SHAP dot plot (B) of fold 1. (A) shows the mean absolute SHAP values for the top 10 essential features in the first cross-validation fold. SHAP values gauge feature importance by showing each feature’s contribution to predictions. Bar length reflects average impact, regardless of direction. This visualization highlights key features in the first fold. (B) through (F) SHAP summary plots (dot plots) for five cross-validation folds. These panels show SHAP summary plots (also known as beeswarm plots) for the top 10 most essential features in each of the five cross-validation folds. The dot’s position indicates the SHAP value: Positive (right of SHAP = 0) pushes the prediction toward the positive class; negative (left) toward the negative class. Dot color indicates feature value, from blue (low) to red (high). Color coding for feature robustness: In (B) to (F), feature name colors (*y* axis labels) indicate their consistency in the top 10 features across five cross-validation folds. Blue: in the top 10 in one and two folds, showing moderate consistency and possible ensitivity. Purple: in three folds, showing good consistency. Red: in four to five folds, indicating high robustness and dependability.

The SHAP bar plot (Fig. 7A, fold 1) ranks features by their mean absolute SHAP value, offering a clear overview of the top 10 features with the greatest impact on model predictions. The analysis highlights several key patterns: (i) Large artery length changes (ΔAmLen_R89) dominate predictions, with increased vessel length consistently linked to positive outcomes; (ii) arterial tortuosity changes show strong predictive power, ranking second in importance; (iii) small-caliber vessel features, particularly changes in smaller vessel segments, display moderate but complex associations with treatment response; and (iv) LOT demonstrates an inverse correlation, where earlier treatment lines contribute positively to favorable predictions.

Cross-validation analysis confirmed high feature stability, with the most predictive features consistently showing importance across all five folds (Fig. 7, B to F). This SHAP analysis offers interpretable insights into which QVMFs and clinical variables are most influential in predicting treatment response. Features that appear across multiple folds (red labels) are likely to be strong biomarkers, which could be helpful in clinical decision-making. The direction of SHAP values (positive versus negative) indicates whether high or low feature values are associated with favorable treatment outcomes, providing practical insights for patient stratification and treatment planning.

#### Individual predictions

**Fig. 8.**
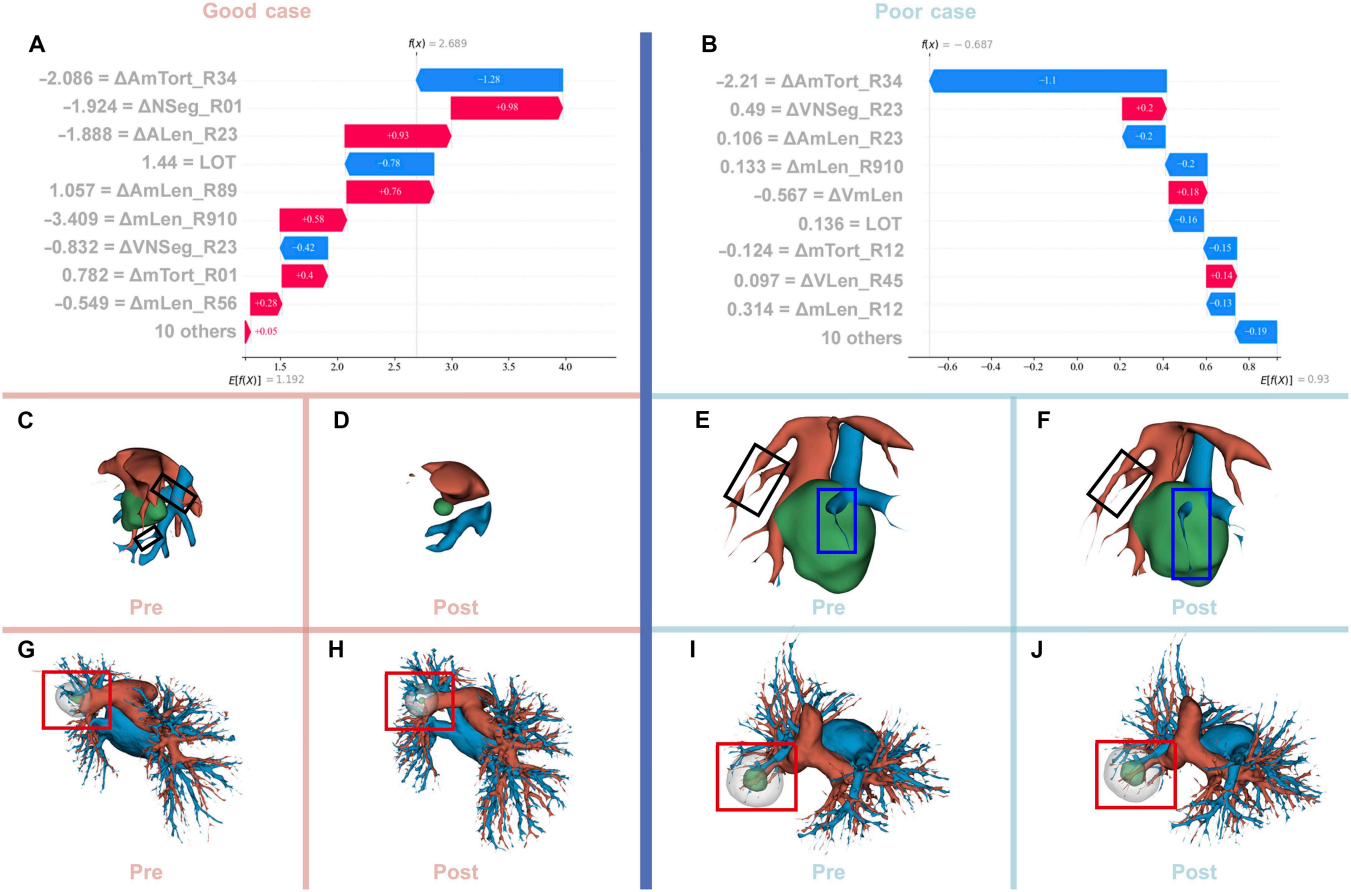
Individual prediction analysis with SHAP waterfall plots and 3D vascular reconstruction. Top row (**A** and **B**): SHAP waterfall plots showing how the top 10 contributing features combine to generate the model’s prediction for each individual patient. Patient-level SHAP waterfall plots for all cases in the internal cross-validation and external validation cohorts are provided as a compressed folder in the supplementary materials. Middle row (**C** to **F**): Magnified 3D views of vessels within the tumor and a 15-mm peritumoral region used for QVMF extraction, before (Pre) and after (Post) anti-angiogenic therapy. These panels depict the local vascular architecture from which length, radius, tortuosity, and end point features are derived. In the responder, peritumoral vessels marked by black boxes show abruptly tapered, attenuated segments before treatment—indicating compression or increased permeability but become smoother and more uniformly opacified after therapy, suggesting improved perfusion and decompression. In contrast, in the nonresponder, vessels highlighted by blue boxes appear as abruptly tapered, thread-like segments both before and after treatment, with little change in vessel caliber or filling, indicating persistent perfusion impairment and abnormal permeability. Bottom row: 3D reconstructions of the tumor, peritumoral zone (translucent gray 15-mm shell), and whole-lung vasculature, with Pre and Post states displayed for each case.

To give a detailed view of the model’s decision process, we examined SHAP waterfall plots for representative cases from the validation set (Fig. 8). Case 1 (good case, the left, pink red): A 42-year-old female with metastatic lung adenocarcinoma carrying epidermal growth factor receptor (EGFR) exon 19 deletion and subsequent T790M [Thr790→Met (T790M)] mutations received sequential targeted therapy culminating in EGFR-TKI plus bevacizumab. The model correctly predicted a favorable response with *f*(*x*) = 2.689, substantially above baseline expectation. Despite some negative contributors, positive vascular features dominated the prediction. The analysis demonstrates that effective anti-angiogenic therapy involves both vascular pruning of abnormal small-caliber vessels and structural recovery of larger vessels. Vascular images show marked structural improvements with vessel regularization and improved organization posttreatment. Clinical validation showed a prolonged PFS of 13 months, confirming the model’s accurate prediction.

Case 2 (poor case, light blue): A 64-year-old female with advanced left upper lobe adenocarcinoma harboring EGFR exon 20 insertion mutation received EGFR-TKI followed by bevacizumab addition. The model correctly predicted poor response with *f*(*x*) = −0.687, substantially below baseline expectation. The most notable negative contributor was arterial tortuosity changes, indicating severely impaired arterial remodeling. Vascular reconstruction images demonstrate minimal structural changes between pre- and posttreatment scans, with persistent irregular vessel architecture consistent with treatment resistance. The clinical outcome confirmed the prediction, with PFS of only 3 months.

## DISCUSSION

Our findings complement and extend previous work on vascular biomarkers in lung cancer therapy (*11*). Further, this study establishes a vascular-centered paradigm for early assessment of anti-angiogenic therapy response in lung cancer by quantifying dynamic changes in tumor and peritumoral vessels on standard-of-care CT imaging. By integrating delta QVMFs with an interpretable machine learning framework, our approach delivers both strong predictive performance and hypothesis-generating insights into treatment-induced vascular remodeling. The superior performance of dynamic QVMFs over static baseline measurements validates the fundamental hypothesis that treatment-induced vascular remodeling contains critical predictive information. This aligns with the vascular normalization theory (*21*, *22*), which posits that effective anti-angiogenic therapy temporarily normalizes aberrant tumor vasculature before regression. Our quantitative approach captures this dynamic process within the critical 4- to 6-week window, providing a potential clinically deployable biomarker that outperforms static snapshots.

The multivariate analysis identified LOT as a robust independent predictor, and earlier treatment lines were significantly associated with better PFS. This aligns with clinical experience, where heavily pretreated tumors often develop resistance mechanisms and complex vascular phenotypes. In our model, LOT acts as a critical clinical context: Early-line tumors likely have a more “plastic” vascular bed that is more responsive to anti-angiogenic modulation. The integration of LOT with delta QVMFs in our delta-merge model suggests that the magnitude of vascular remodeling is constrained by the tumor’s treatment history, emphasizing the importance of deploying anti-angiogenic strategies early in the therapeutic sequence to maximize the potential for vascular normalization.

The analysis identified specific QVMFs’ changes that distinguish responders from nonresponders, aligning with the mechanism of vascular pruning (*21*–*24*). In the responder group, we observed a significant reduction in vessel end points (Fig. 3D, *P* < 0.01) and a trajectory toward simplified vascular states (Fig. 3E). This “pruning” likely represents the selective elimination of immature, inefficient, and leaky vessels while preserving functional hierarchical structures, a hallmark of vascular normalization. In contrast, nonresponders exhibited network deterioration or chaotic persistence. The ability of our automated pipeline to quantify this structural simplification supports the concept that effective therapy does not merely destroy vessels. Still, it remodels the network into a more efficient, less tortuous architecture, optimizing perfusion for therapeutic delivery.

A notable finding from our interpretable model was the dominant predictive value of large arterial features, specifically the elongation of arterial segments in the 8- to 9-mm radius bin (Fig. 7; purple, ΔAmLen_R89; blue, ΔVmLen_R89). We propose a “hilar vascular decompression” hypothesis to explain this phenomenon. A substantial proportion of advanced lung cancers are central or have heavy hilar/mediastinal disease burden, often compressing or invading adjacent pulmonary artery branches. In responders, tumor shrinkage alleviates mechanical compression, allowing these large vessels to reexpand and recover their natural morphology, which our algorithm detects by increasing vessel length and regularity. Conversely, in nonresponders, persistent tumor bulk maintains vascular compression or invasion, associated with poor prognosis. Thus, the high weight assigned to these large-vessel features suggests that our model effectively captures the reversal of high-risk anatomical compromises—specifically, the liberation of major pulmonary vessels—thereby serving as a robust surrogate for primary tumor regression in centrally located disease.

While large arteries reflected structural recovery, Tortuosity of small-caliber vessels (Fig. 7; red, ΔAmTort_R34; purple, ΔmTort_R12; blue, ΔmTort_R01) displayed an adaptive pattern that, at first glance, appears paradoxical: Responders maintained or even increased specific tortuosity metrics compared to nonresponders, so did ΔVNSeg_R23 (Fig. 7, red). We attribute this to perfusion recovery driven by vascular maturation. In nonresponders, rapid tumor progression and elevated interstitial fluid pressure (IFP) likely lead to the collapse and necrosis of the small-caliber vessels. In responders, however, anti-angiogenic therapy promotes vascular wall repair and reduces vascular permeability (*8*). This structural normalization lowers IFP and restores perfusion to previously nonfunctional, collapsed vessels, making them visible again on contrast-enhanced CT. Therefore, the “preservation” or relative increase in small-vessel tortuosity in the good-prognosis group should be interpreted not as pathological angiogenesis but as the reopening and functional restoration of the small-caliber vessel bed, facilitating better drug delivery and oxygenation (*2*).

By integrating delta QVMFs derived from standard-of-care CT, which quantify treatment-induced changes in vascular morphology within tumor and peritumoral vessels, with an interpretable machine learning framework, our approach delivers both strong predictive performance and hypothesis-generating insights into treatment-related vascular remodeling. These findings are consistent with the vascular normalization theory proposed by Jain *et al.*, which posits that effective anti-angiogenic therapy transiently normalizes abnormal tumor vasculature before subsequent regression (*21*, *22*). They also echo the vessel architectural imaging study by Emblem *et al.* (*25*), in which anti–vascular endothelial growth factor therapy–induced shifts toward smaller-caliber, more organized, and better-oxygenated microvessels were associated with improved progression-free and overall survival. In our cohort, delta QVMFs captured treatment-induced vascular changes approximately 4 to 6 weeks after initiation of anti-angiogenic therapy, suggesting that CT-based vascular morphometry may provide an early, noninvasive biomarker for response assessment and treatment decision-making. Notably, whereas Emblem *et al.* probed microvascular calibers at the MRI perfusion scale, our QVMFs are derived from millimeter-scale CT voxels and primarily reflect the geometry and visibility of small arterial and venous segments. The apparent increase in specific small-vessel radius bins observed in responders may therefore largely reflect improved contrast filling and reduced leakage—allowing previously poorly visualized vessels to be segmented more clearly—rather than true structural dilation. Viewed together, these results support the notion that successful anti-angiogenic therapy manifests as scale-dependent vascular normalization, which can be captured by both advanced MRI and routine CT–based vascular morphometry.

The importance of interpretable machine learning in precision medicine has been increasingly recognized, particularly for high-stakes medical decisions (*18*, *26*–*28*). The SHAP analysis integrates these findings into a coherent arterial-dominant, venous-adaptive vascular signature. Beyond cohort-level patterns, the SHAP waterfall plots (Fig. 8, A and B) provide a granular view of individual decision-making, allowing clinicians to trace specific feature contributions back to the patient’s corresponding CT images. By transparently visualizing how large-vessel structural recovery (decompression) synergizes with small-caliber vessels’ functional restoration (perfusion), the model moves beyond black-box predictions to provide anatomically meaningful evidence. The feature correlation networks (Fig. 5) further illustrate that responders maintain a coordinated, modular vascular phenotype distinct from the disorganized connectivity seen in nonresponders.

This study provides the potential clinical applicability of QVMFs derived from standard-of-care CT to guide personalized anti-angiogenic therapy in lung cancer. The model is interpretable, with SHAP elucidating feature contributions at both cohort and patient levels, thereby enhancing clinical trust in decision support. Robust performance was maintained in an external validation cohort despite substantial differences in imaging parameters, including arterial-phase acquisition and increased slice thickness, as well as across heterogeneous treatment regimens. Consistent results in a dedicated subgroup receiving standard anti-angiogenic therapy combined with chemotherapy and immunotherapy further support the robustness of the approach. Notably, the fully automated pipeline enables practical integration of QVMFs into routine clinical workflows.

### Limitations

Several limitations should be acknowledged. This was a retrospective, two-center study, with model development and internal validation at a single cancer center and external validation at one additional tertiary hospital. Patient characteristics, treatment patterns, and imaging protocols reflect a limited institutional spectrum, so larger prospective multicenter studies are needed to confirm generalizability. Model training and internal validation used venous-phase CT images, while external validation used arterial-phase imaging. This mismatch in contrast phase may affect QVMF characterization, especially for small vessels, and could bias feature distributions across cohorts. Future studies with harmonized multiphase imaging or phase-specific strategies, ideally within a prospective multicenter design, may address these limitations.

Second, imaging was performed at a single early follow-up time point and did not explicitly model the dose- and time-dependent vascular normalization window induced by anti-angiogenic therapy (*29*). Given heterogeneous dosing regimens and treatment schedules in this real-world cohort, scans may have captured different stages of vascular remodeling, potentially attenuating observed associations. Prospective studies with standardized dosing, controlled imaging time points, and patient-specific modeling of normalization dynamics are warranted. Last, although SHAP-based analyses provide interpretable insights into feature contributions at the patient level, these imaging-derived findings remain hypothesis-generating and require validation against clinical, histopathologic, and molecular correlates to confirm the proposed biological mechanisms.

## MATERIALS AND METHODS

### Experimental design

**Fig. 9.**
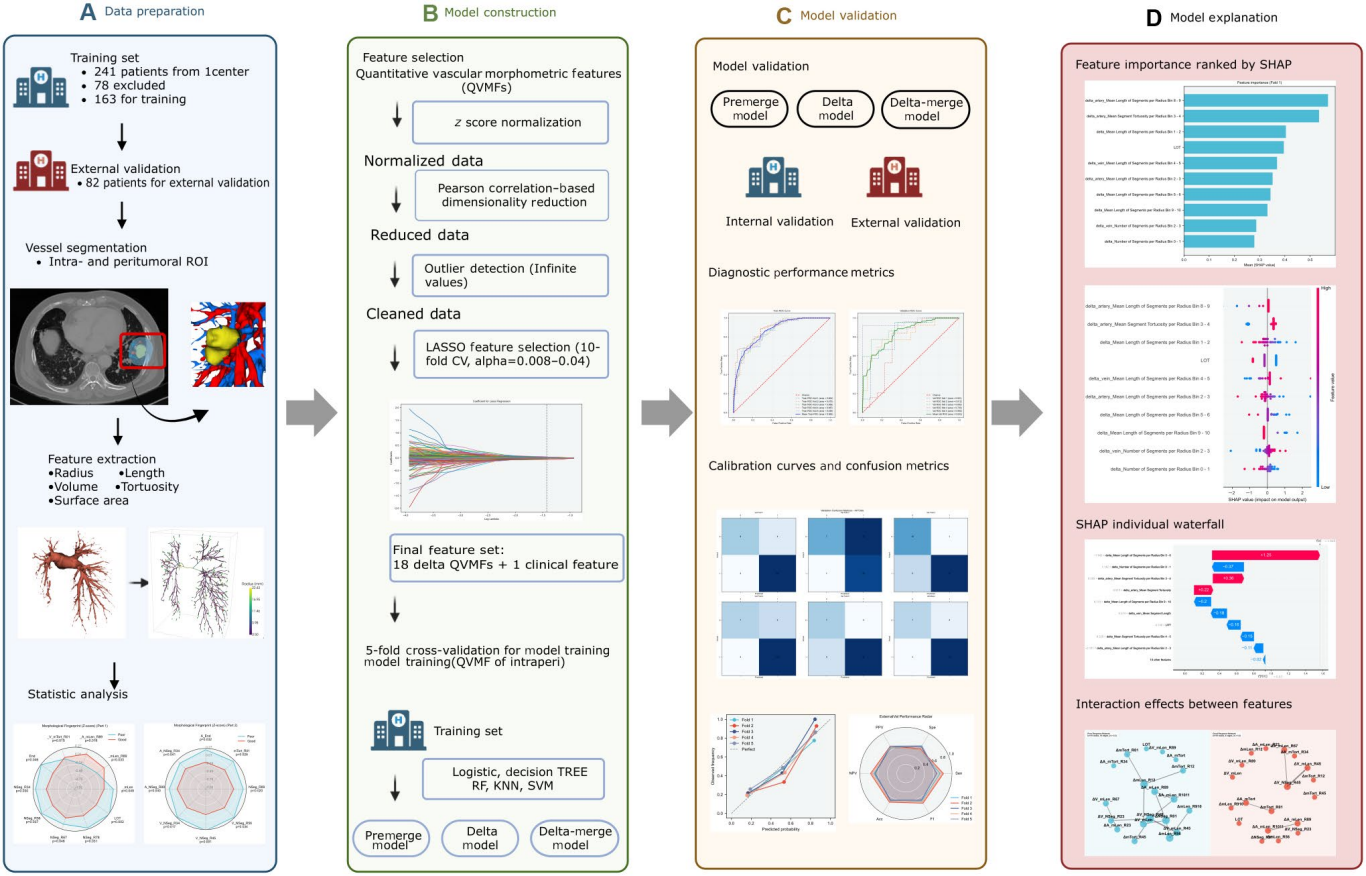
Outline of the study. Study workflow comprising four sections: (**A**) Data preparation (patient enrollment, vessel segmentation, feature extraction, and statistic analysis); (**B**) model construction (feature selection via normalization, dimensionality reduction, LASSO selection resulting in 18 delta QVMFs and 1 clinical feature, followed by fivefold CV training using multiple ML algorithms to generate premerge, Delta, and Delta-merge models); (**C**) model validation (performance evaluation with ROC curves, calibration curves, and performance metrics); and (**D**) model explanation (SHAP-based feature importance, summary plots, waterfall plots, and feature interaction networks).

This retrospective cohort study was designed to address the marked heterogeneity in clinical response to anti-angiogenic therapy in lung cancer. The outline of the study is shown in Fig. 9. We developed and validated an interpretable machine learning framework using dynamic QVMFs derived from standard-of-care contrast-enhanced CT imaging.

To capture early treatment-induced vascular remodeling, comprehensive QVMFs were extracted from baseline and first follow-up scans acquired 4 to 6 weeks after therapy initiation. Delta changes in vascular morphology were explicitly modeled rather than relying solely on static baseline features. We hypothesized that dynamic vascular remodeling patterns would provide superior predictive performance for assessing early treatment response.

### Patient population

This study was approved by the Institutional Review Boards (IRBs) of Hubei Cancer Hospital (No. LLHBCH2025YN-055) and the Ethics Committee of the external validation center (No. 2024-S048). Both IRBs waived the requirement for informed consent due to the study’s retrospective nature. We identified a cohort of patients with advanced lung cancer who received treatment between January 2020 and May 2025. Inclusion criteria were as follows: (i) pathologically confirmed lung cancer, (ii) received at least one cycle of anti-angiogenic therapy, (iii) had available contrast-enhanced CT scans before therapy start (baseline) and during the first follow-up assessment (typically 4 to 6 weeks after therapy initiation), and (iv) had measurable lesions according to RECIST 1.1. Exclusion criteria were (i) absence of adequate quality CT images, (ii) lesion too small to be delineated by used AI tools, (iii) PFS follow-up data less than 6 months, (iv) extensive pulmonary conditions that substantially affect vascular distribution and segmentation accuracy (e.g., extensive bullae or severe emphysema affecting >50% of lung volume, massive atelectasis, bilateral pneumothorax), or pulmonary conditions affecting the tumor or peritumoral region that compromise vascular segmentation (e.g., bullae or emphysema in the tumor or peritumoral region, pneumothorax on the tumor-bearing side, extensive pneumonia, or interstitial pneumonia affecting the peritumoral region), or other conditions that compromise accurate vessel segmentation and feature extraction in the region of interest. We included 163 patients in the final analysis for the primary cohort and 82 patients for the external validation cohort.

### Clinical data and imaging

Clinical data, including age, sex, and tumor histology, were collected from electronic medical records. For the training and internal validation cohorts, all patients underwent contrast-enhanced chest CT on 64- or 128-detector scanners (GE Healthcare or Siemens Healthineers) using standardized protocols: tube voltage of 120 kVp, slice thickness of 0.625 or 1.0 mm, and intravenous administration of 80 to 100 ml of contrast material at a rate of 1.8 to 3.0 ml/s. Venous-phase images, acquired 35 s after the arterial phase, were used for model training and internal validation. Baseline and first follow-up scans, typically obtained 4 to 6 weeks after treatment initiation, were analyzed to capture early treatment-induced vascular changes.

For the external validation cohort, contrast-enhanced chest CT scans were acquired using arterial-phase imaging with slice thicknesses of 1.5 or 2.0 mm, reflecting routine clinical acquisition protocols at the external institution. Despite differences in imaging phase and spatial resolution relative to the training data, the same preprocessing, vascular segmentation, and feature extraction pipeline was applied to all datasets, except that external images were resampled to 1 or 0.625 mm to ensure consistency across cohorts.

### Vascular feature extraction

Vascular networks and three-dimensional (3D) tumor ROIs were automatically segmented from venous-phase images using a validated deep learning–based software (uAI Pioneer Portal v1.0), and quantitative vascular morphometric features were computed using VesselVio v1.1 (*30*). Two radiologists, blinded to outcomes, reviewed the segmentations and 3D tumor ROIs with a 15-mm peritumoral margin. For each patient, we derived (i) pretreatment features from baseline scans, including segment-level (length, tortuosity, radius), network-level (endpoints, branch counts), and radius-binned metrics (e.g., 0–1, 1–2, 2–3 mm, etc.). The overall view of QVMFs is shown in fig. S4, and (ii) delta features, defined as posttreatment minus pretreatment values, capture treatment-induced vascular remodeling. Vessel tortuosity was calculated from the 3D centerlines as the ratio of centerline path length to the Euclidean distance between end points, providing a quantitative but not directly visual tortuosity measure on raw CT images. All vascular morphometric features (radius, length, tortuosity, volume, and surface area, and their radius-binned derivatives) were therefore imaging-derived surrogates computed from CT-based segmentation masks and voxel geometry and should not be interpreted as exact anatomical diameters or lengths. For clarity, all vascular morphometric features are denoted using standardized abbreviations (e.g., ΔmLen, ΔAmLen_R89, and VNSeg_R45); a complete list of abbreviations and their definitions is provided in table S7.

### Outcome definition and statistical analysis

We defined the primary end point as PFS, calculated as the time from the start of anti-angiogenic therapy to disease progression. We assessed disease progression according to RECIST 1.1. We dichotomized patients into two groups for binary classification: a poor-prognosis group (label = 0, PFS < 6 months) and a good-prognosis group (label = 1, PFS ≥ 6 months).

Statistical analyses, univariate and multivariate analysis screening (*P* < 0.05), were performed using Python 3.9 (libraries: scipy, statsmodels, scikit-learn). Continuous variables were compared using Student’s *t* test (for normally distributed data) or Mann-Whitney *U* test (for nonnormally distributed data). Nominal categorical variables were analyzed using the Chi-square test, while ordinal variables (e.g., ECOG, LOT) were evaluated using the Mann-Whitney *U* test to detect shifts in distribution. Model performance was evaluated using the AUC, estimated from cross-validation. Confidence intervals for AUC were not calculated, as performance was assessed across repeated resampling rather than as a single population estimate.
