# Supplementary material for: Interpretable dynamic quantitative vascular morphometry features using SHAP for anti-angiogenic therapy response prediction
Source: Sci Adv. 2026 Jul 24;12(30):eaeb3543. doi: 10.1126/sciadv.aeb3543 (PMC13398484; doi:10.1126/sciadv.aeb3543)
Supplement: Supplementary file 1 — Figs. S1 to S4 Tables S1 to S7 Legends for data S1 and S2 [file sciadv.aeb3543_sm.pdf]

Science Advances
AAAS

# Supplementary Materials for

## Interpretable dynamic quantitative vascular morphometry features using SHAP for anti-angiogenic therapy response prediction

Kui Hu *et al.*

Corresponding author: Wuling Ou, 609700817@qq.com; Yulin Liu, liuyl26@163.com

*Sci. Adv.* **12**, eaeb3543 (2026)
DOI: 10.1126/sciadv.aeb3543

**The PDF file includes:**

Figs. S1 to S4
Tables S1 to S7
Legends for data S1 and S2

**Other Supplementary Material for this manuscript includes the following:**

Data S1 and S2

**Fig. S1.**

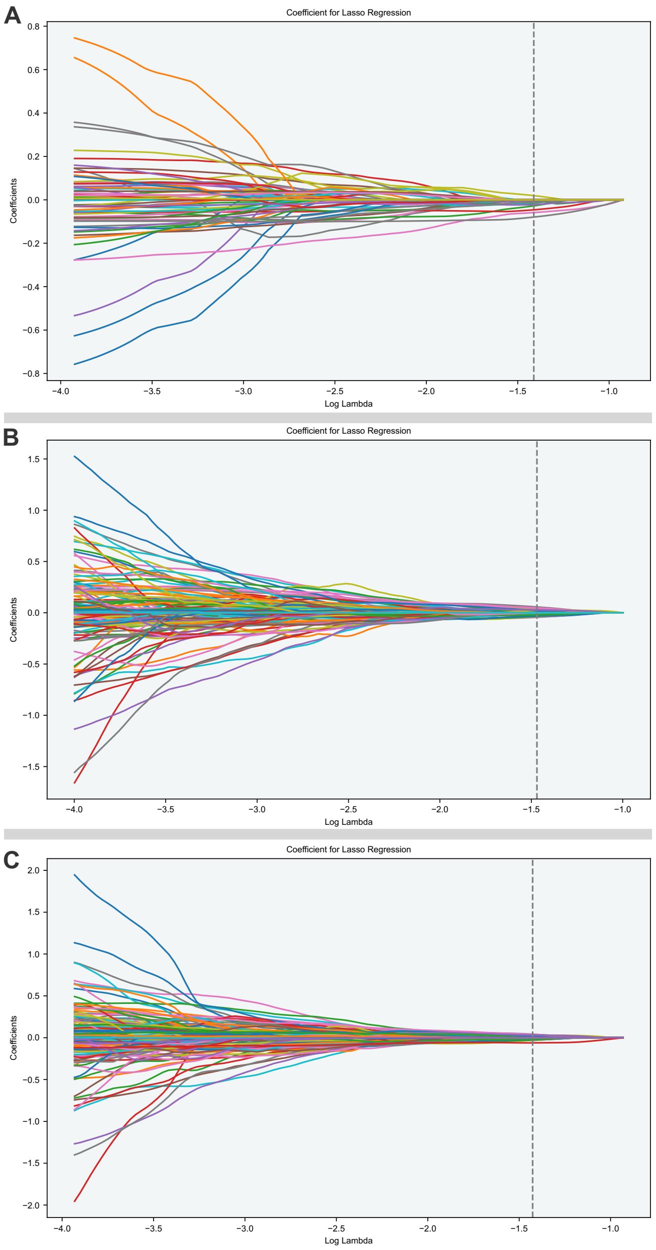


**Figure S1. LASSO regression plots for three models.**

Panel A-C corresponds to the Pre-merge, Delta, and Delta-merge models

## Fig. S2.

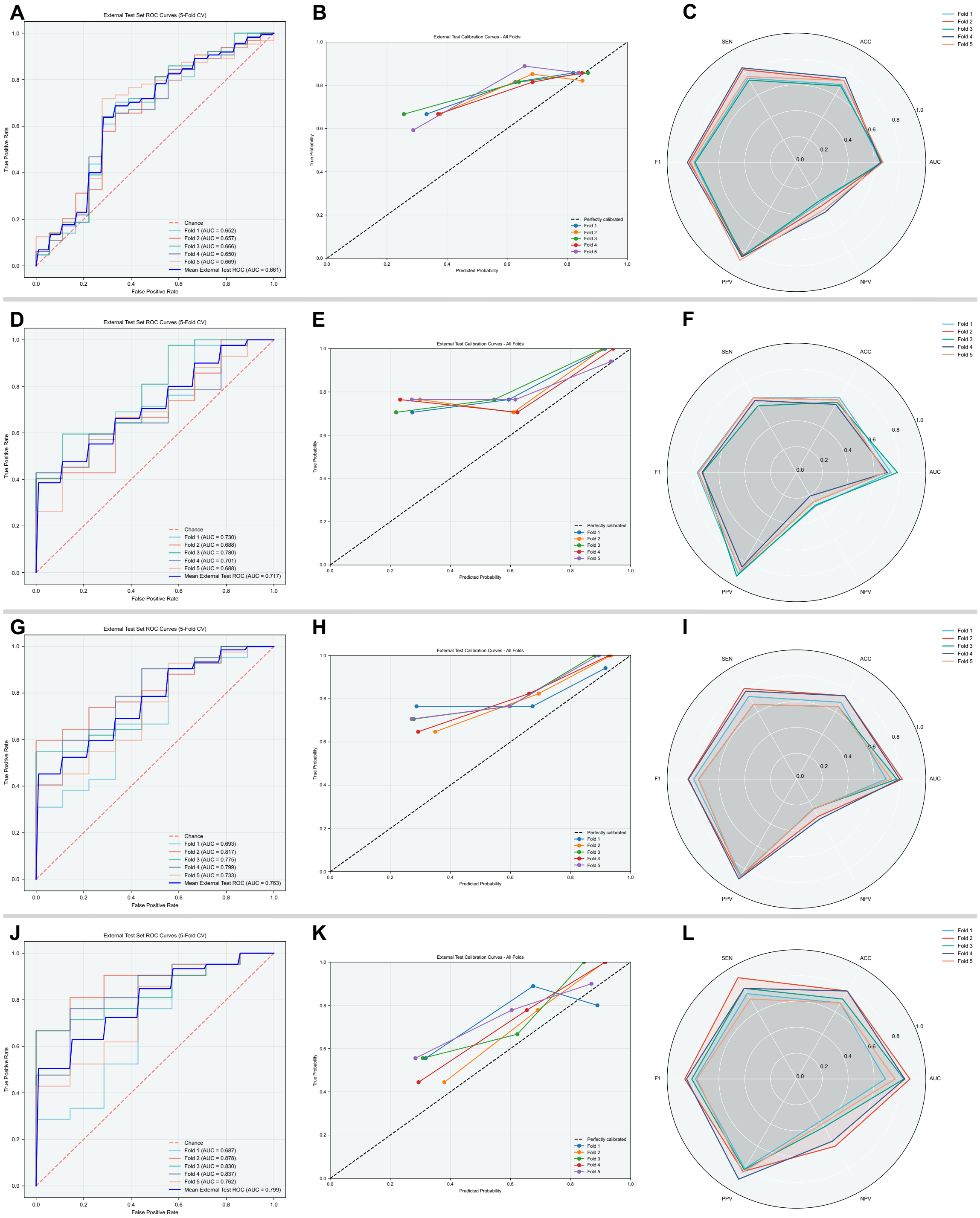


**Figure S2. External Validation and Subgroup Performance of Pre-Merge, Delta, and Delta-Merge Models**

Panels A–C display ROC, calibration curves, and performance radar for the Pre-Merge model (A, average AUC =0.661; C, average NPV = 0.393). D–F show these for the Delta model (D, average AUC =0.717; F, average NPV = 0.266). Panels G–I

present the same for the Delta-Merge model (G, average AUC = 0.763, average NPV = 0.297). Panel J–L presents the same for the Delta-Merge model on the subset (J, average AUC =0.799; L, average NPV = 0.472).

**Fig. S3.**

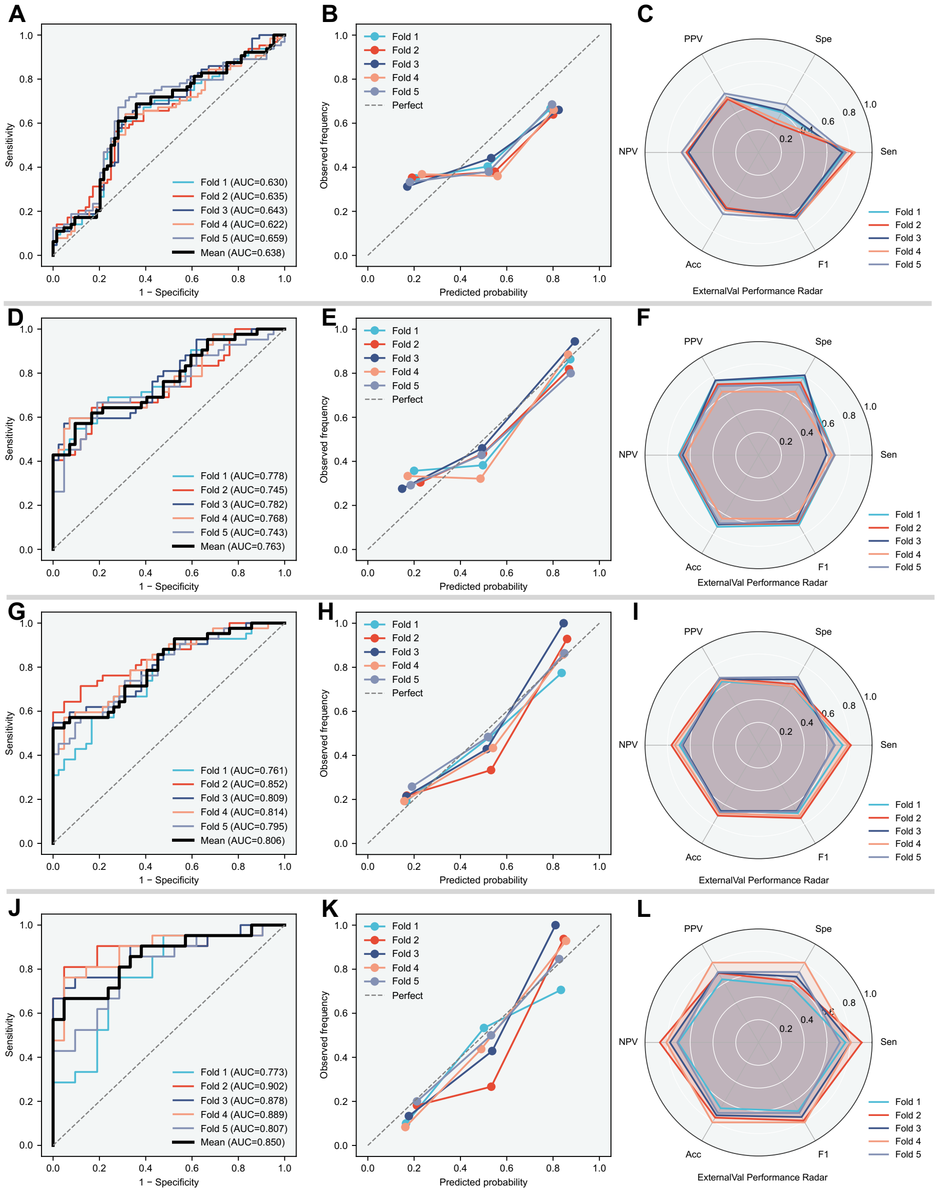


**Figure S3: External Validation and Subgroup Performance of Pre-Merge, Delta, and Delta-Merge Models with SMOTE.**

Panels A–C display ROC, calibration curves, and performance radar for the Pre-Merge model (A, average AUC =0.638; C, average NPV = 0.643). D–F show these for the Delta model (D, average AUC =0.763; F, average NPV = 0.676). Panel G–I presents the same for the Delta-Merge model (G, average AUC =0.806; I, average NPV = 0.707). Panel J–L presents the same for the Delta-Merge model on subset (J, average AUC =0.850; L, average NPV = 0.775).

**Fig. S4.**

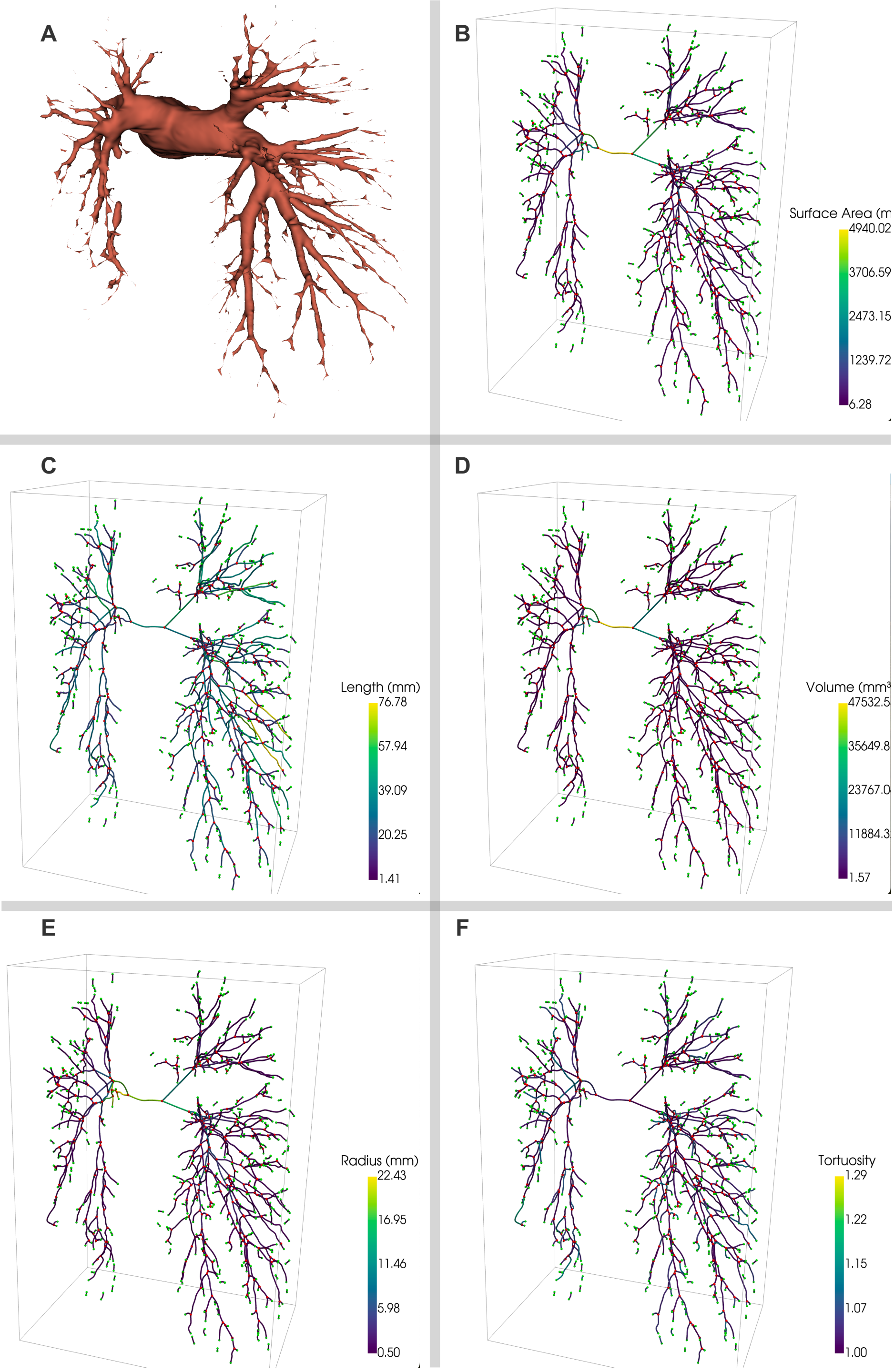


**Figure S4. Overall view of QVMFs extraction from the 3D vessel structure.**

Panel A represents a 3D artery vessel. Panel B-F corresponds to vascular Surface Area ($mm^2$), Length (mm), Volume ($mm^3$), Radius (mm), and Tortuosity.

**Table S1.**

**Clinical characteristics of patients stratified by treatment response from external validation.**

| Characteristic | Poor Response (n = 18) | Good Response (n = 64) | P value |
|---|---|---|---|
| Age (years) | 58.11 ± 9.37 | 60.11 ± 9.18 | 0.470 |
| BMI (kg/m²) | 23.07 ± 3.98 | 22.87 ± 2.98 | 0.856 |
| LDH | 212.61 ± 52.17 | 229.09 ± 97.01 | 0.602 |
| Sex | | | 1.000 |
| Female | 7 (38.9%) | 26 (40.6%) | |
| Male | 11 (61.1%) | 38 (59.4%) | |
| Histology | | | 0.414 |
| Adenocarcinoma | 17 (94.4%) | 60 (93.7%) | |
| Squamous cell carcinoma | 0 (0.0%) | 3 (4.7%) | |
| Small cell carcinoma | 1 (5.6%) | 1 (1.6%) | |
| Line of therapy (LOT) | | | 0.260 |
| First-line | 10 (55.6%) | 48 (75.0%) | |
| Second-line | 6 (33.3%) | 11 (17.2%) | |
| Third-line or later | 2 (11.1%) | 5 (7.8%) | |
| Treatment category (CCT) | | | 0.057 |
| Chemotherapy | 1 (5.5%) | 6 (9.4%) | |
| TKI | 2 (11.1%) | 24 (37.5%) | |
| Monotherapy | 3 (16.7%) | 2 (3.1%) | |
| Immunotherapy | 12 (66.7%) | 30 (46.9%) | |
| Others | 0 (0.0%) | 2 (3.1%) | |

Note: P values were calculated using the Mann–Whitney U test for continuous variables and the chi-square for categorical variables, as appropriate.

The external validation cohort included 82 patients; all were used to validate the pre-merge model, whereas 51 patients with both baseline and follow-up imaging were included in the delta and delta-merge model validations. Subgroup analysis of patients receiving the standard combination of anti-angiogenic therapy, chemotherapy, and immunotherapy was performed in a subset of 28 patients. Due to class imbalance in the external validation cohort, a resampling strategy based on the

synthetic minority over-sampling technique (SMOTE) was used to facilitate model evaluation.

**Table S2.**

**Univariable and multivariable analyses of clinical and QVMFs associated with treatment response.**

| Feature | Label 0 (Mean ± SD) | Label 1 (Mean ± SD) | Univariable P value | Multivariable P value (OR (95% CI), low-high) |
|---|---|---|---|---|
| ΔmLen | 0.45 ± 2.27 | -0.27 ± 2.70 | 0.0491 | 0.87(0.76-1.00), 0.0493 |
| ΔmLen_R89 | -1.05 ± 5.16 | 0.25 ± 4.76 | 0.0331 | — |
| ΔAmLen_R89 | -1.33 ± 4.16 | 0.21 ± 4.81 | 0.0158 | — |
| ΔVmTort_R01 | -0.04 ± 0.35 | -0.03 ± 0.28 | 0.0148 | — |
| LOT | 2.13 ± 0.73 | 1.75 ± 0.76 | 0.0020 | 0.54 (0.34–0.85), 0.0075 |
| End | 45.46 ± 29.72 | 34.71 ± 19.74 | 0.0460 | — |
| NSeg_R34 | 6.75 ± 7.21 | 4.53 ± 6.48 | 0.0300 | — |
| NSeg_R56 | 1.79 ± 2.73 | 0.84 ± 1.91 | 0.0375 | — |
| NSeg_R67 | 0.90 ± 1.42 | 0.61 ± 1.48 | 0.0459 | — |
| NSeg_R78 | 0.68 ± 1.37 | 0.26 ± 0.82 | 0.0307 | — |
| NSeg_R89 | 0.38 ± 0.85 | 0.16 ± 0.65 | 0.0199 | — |
| mTort_R01 | 1.06 ± 0.08 | 1.04 ± 0.05 | 0.0264 | $3.87 \times 10^{-5}$ ($7.09 \times 10^{-9}$–0.211), 0.0206 |
| AEnd | 23.92 ± 16.21 | 18.44 ± 12.34 | 0.0320 | — |
| ANSeg_R34 | 2.81 ± 3.21 | 2.03 ± 3.06 | 0.0411 | — |
| ANSeg_R89 | 0.32 ± 0.89 | 0.14 ± 0.57 | 0.0395 | — |
| VNSeg_R34 | 2.33 ± 3.35 | 1.18 ± 2.08 | 0.0167 | — |
| VNSeg_R45 | 1.35 ± 2.29 | 0.45 ± 1.29 | 0.0014 | — |
| VNSeg_R56 | 0.67 ± 1.52 | 0.20 ± 0.68 | 0.0340 | — |

**Table S3.**

**Paired t-test analysis of pre- and post-treatment QVMFs.**

**A. Arterial combined Venous QVMFs**

| Feature | P value (Overall) | P value (Good) | P value (Poor) | Mean (Pre) | Mean (Post) | Change |
|---|---|---|---|---|---|---|
| End | 0.00000693 | 0.00047065 | 0.00478907 | 38.87 | 34.20 | Decrease |
| SurfArea | 0.00000976 | 0.00001450 | 0.05353237 | 8303.01 | 6853.09 | Decrease |
| Vol | 0.00001340 | 0.00001150 | 0.07967024 | 13202.29 | 10406.21 | Decrease |
| NetLen | 0.00001680 | 0.00016430 | 0.01886370 | 619.71 | 531.41 | Decrease |
| NSeg | 0.00024766 | 0.00934118 | 0.01067311 | 55.72 | 48.17 | Decrease |
| Branch | 0.00242276 | 0.03389100 | 0.02856447 | 23.61 | 20.12 | Decrease |
| mVol | 0.01486080 | 0.00362792 | 0.56062134 | 175.20 | 160.63 | Decrease |
| mRad | 0.02486173 | 0.13295565 | 0.07620535 | 1.75 | 1.67 | Decrease |
| mSurfArea | 0.03849337 | 0.02283577 | 0.64645753 | 126.60 | 121.94 | Decrease |
| mTort_R01 | 0.37357551 | 0.62294500 | 0.02749302 | 1.05 | 1.05 | Decrease |
| NSeg_R67 | 0.00571043 | 0.03160119 | 0.07372758 | 0.72 | 0.48 | Decrease |
| NSeg_R56 | 0.00610264 | 0.09861264 | 0.02131792 | 1.21 | 0.88 | Decrease |
| NSeg_R45 | 0.00162340 | 0.00191956 | 0.14696139 | 2.71 | 2.06 | Decrease |
| NSeg_R34 | 0.00728723 | 0.09593080 | 0.02728365 | 5.39 | 4.48 | Decrease |
| NSeg_R23 | 0.02237849 | 0.27590611 | 0.02366365 | 11.09 | 9.40 | Decrease |
| NSeg_R12 | 0.00493349 | 0.09808884 | 0.01478109 | 20.104 | 17.50 | Decrease |
| NSeg_R01 | 0.04036288 | 0.01330848 | 0.81436617 | 13.34 | 12.40 | Decrease |
| NSeg_R910 | 0.08387152 | 0.01393182 | 0.77351161 | 0.29 | 0.16 | Decrease |

**B. Arterial QVMFs**

| Feature | P value (Overall) | P value (Good) | P value (Poor) | Mean (Pre) | Mean (Post) | Change |
|---|---|---|---|---|---|---|
| ANSeg | 0.00331242 | 0.02098921 | 0.05995142 | 25.67 | 22.05 | Decrease |
| AEnd | 0.00538111 | 0.02160355 | 0.12371510 | 20.56 | 18.31 | Decrease |
| ANSeg_R12 | 0.00547161 | 0.02847790 | 0.08052828 | 8.94 | 7.60 | Decrease |
| ANSeg_R910 | 0.02795788 | 0.02347739 | 0.33211198 | 0.19 | 0.08 | Decrease |

**C. Venous QVMFs**

| Feature | P value (Overall) | P value (Good) | P value (Poor) | Mean (Pre) | Mean (Post) | Change |
|---|---|---|---|---|---|---|
| VmVol | 0.00071990 | 0.00156014 | 0.12855242 | 165.05 | 141.73 | Decrease |
| VNSeg_R23 | 0.00133006 | 0.11343501 | 0.00207507 | 4.04 | 3.18 | Decrease |
| VmSurfArea | 0.00158749 | 0.00106305 | 0.28552401 | 140.93 | 127.36 | Decrease |
| VEnd | 0.00206753 | 0.00299773 | 0.15284905 | 18.26 | 16.10 | Decrease |
| VNSeg | 0.00393923 | 0.03344302 | 0.04596906 | 21.25 | 18.34 | Decrease |
| VNSeg_R12 | 0.01128649 | 0.02563368 | 0.14637028 | 8.17 | 7.06 | Decrease |
| VNSeg_R45 | 0.01359324 | 0.65932524 | 0.00423202 | 0.80 | 0.56 | Decrease |
| VmRad | 0.01562638 | 0.24239137 | 0.01784822 | 1.57 | 1.51 | Decrease |
| VSegPart | 0.02496864 | 0.00786467 | 0.71161377 | 0.08 | 0.09 | **Increase** |
| VmLen | 0.02946077 | 0.00778480 | 0.86948987 | 13.38 | 12.54 | Decrease |
| VNSeg_R56 | 0.03058740 | 0.58329345 | 0.02448396 | 0.38 | 0.24 | Decrease |
| VNSeg_R67 | 0.10405924 | 0.02435792 | 0.80457095 | 0.19 | 0.12 | Decrease |

Note: Mean values are reported for pre- and post-treatment measurements. Features are grouped into global, arterial, and venous categories. Change indicates the direction of feature variation from pre- to post-treatment.

Paired analysis of pre- and post-treatment imaging demonstrated widespread vascular remodeling following therapy, with significant reductions in global network-level features, including vessel endpoints, surface area, volume, and segment number (Supplementary Tables S3 A-C). Compartment-specific analysis revealed consistent decreases in both arterial and venous metrics, with more extensive and statistically robust changes observed in the venous vasculature. These quantitative alterations provide a structural basis for the imaging biomarkers subsequently incorporated into the predictive models.

**Table S4.**

**LASSO Regression Coefficients for Selected Features Across Three Models.**

***A. Pre-treatment and Clinical Features (Pre-Merge Model)***

| Feature Category | Name | Pre-Merge Model |
|---|---|---|
| | Cut-off | 0.613496933 |
| | Lamda | 0.038707516 |
| Clinical | LOT | -0.079545 |
| Coefficients of pre-treatment features | mLen_R01 | -0.0276226 |
| | Tort_R01 | -0.0461346 |
| | AEnd | -0.0063084 |
| | ANSeg_R01 | -0.0152182 |
| | ANSeg_R78 | -0.0119867 |
| | VmLen | 0.01944307 |
| | VNSeg_R23 | -0.0224866 |
| | VNSeg_R45 | -0.0587734 |
| | VNSeg_R56 | -0.0106689 |

**B. *Dynamic* Vascular Features (delta and delta-Merge Models)**

| Feature Category | Name | delta model | Delta-Merge model |
|---|---|---|---|
| Parameters of LASSO | Cut-off | 0.613496933 | 0.613496933 |
| | lamda | 0.033941929 | 0.037456794 |
| Clinical Characteristics | LOT | | -0.061822725 |
| Coefficients of Vascular features | ΔNSeg_R01 | -0.039375362 | -0.026251589 |
| | ΔmLen_R12 | -0.04382254 | -0.032739654 |
| | ΔmLen_R34 | -0.01197987 | |
| | ΔmLen_R56 | -0.037762672 | -0.031586434 |
| | ΔmLen_R910 | -0.01877921 | -0.021273755 |
| | ΔmTort_R01 | 0.007904917 | 0.001415023 |
| | ΔmTort_R12 | 0.056628075 | 0.041699597 |
| | ΔmTort_R45 | -0.026235557 | -0.016315363 |
| | ΔmTort_R67 | 0.001193699 | |
| | ΔAmTort | | 0.000749654 |
| | ΔAmLen_R23 | -0.034314636 | -0.026244021 |
| | ΔAmLen_R89 | 0.039579722 | 0.031307803 |
| | ΔAmLen_R910 | -0.004842773 | |
| | ΔAmLen_R1011 | 0.010745508 | 0.013156034 |
| | ΔAmTort_R34 | 0.044111489 | 0.029225154 |
| | ΔVmLen | -0.032071936 | -0.020431676 |

| | | | |
|---|---|---|---|
| | ΔVNSeg_R23 | 0.047907928 | 0.038946015 |
| | ΔVNSeg_R45 | 0.022148191 | 0.028690173 |
| | ΔVmLen_R45 | 0.023704521 | 0.016800928 |
| | ΔVmLen_R67 | -0.021497789 | -0.016984849 |
| | ΔVmLen_R89 | 0.028564452 | 0.032232504 |

**Table S5.**

**Machine Learning Algorithm Performance on Internal Validation Summary of The Delta-merge Model.**

| Classifier | AUC | Accuracy | Recall | Precision | F1_score |
|---|---|---|---|---|---|
| Logistic Regression | 0.843 ± 0.088 | 0.761 ± 0.118 | 0.857 ± 0.060 | 0.796 ± 0.206 | 0.808 ± 0.109 |
| Support Vector Machine | 0.774 ± 0.069 | 0.713 ± 0.151 | 0.885 ± 0.092 | 0.734 ± 0.196 | 0.784 ± 0.125 |
| Random Forest | 0.752 ± 0.040 | 0.706 ± 0.088 | 0.881 ± 0.098 | 0.722 ± 0.157 | 0.778 ± 0.082 |
| K-Nearest Neighbors | 0.698 ± 0.107 | 0.681 ± 0.083 | 0.853 ± 0.097 | 0.707 ± 0.142 | 0.760 ± 0.077 |
| Decision Tree | 0.622 ± 0.078 | 0.632 ± 0.070 | 0.696 ± 0.093 | 0.710 ± 0.147 | 0.690 ± 0.075 |

**Table S6.**

**External Validation Performance of Three Model with and without SMOTE**

| Model | | AUC | Accuracy | Recall | Precision | F1 | PPV | **NPV** |
|---|---|---|---|---|---|---|---|---|
| Pre-merge | original | 0.661± 0.008 | 0.720± 0.030 | 0.784± 0.049 | 0.846± 0.018 | 0.813± 0.025 | 0.846± 0.018 | 0.393± 0.041 |
| | SMOTE | 0.638± 0.014 | 0.586± 0.023 | 0.784± 0.049 | 0.563± 0.021 | 0.654± 0.017 | 0.563± 0.021 | **0.643**± 0.030 |
| Delta model | original | 0.717± 0.039 | 0.639± 0.022 | 0.649± 0.031 | 0.885± 0.031 | 0.747± 0.019 | 0.885± 0.031 | 0.266± 0.035 |
| | SMOTE | 0.763± 0.019 | 0.693± 0.031 | 0.648± 0.031 | 0.715± 0.047 | 0.679± 0.025 | 0.715± 0.047 | **0.676**± 0.023 |
| Delta-merge model | original | 0.**763**± 0.050 | 0.694± 0.049 | 0.733± 0.066 | 0.875± 0.011 | 0.797± 0.040 | 0.875± 0.011 | 0.297± 0.045 |
| | SMOTE | 0.**806**± 0.033 | 0.683± 0.020 | 0.733± 0.066 | 0.667± 0.015 | 0.697± 0.030 | 0.667± 0.015 | **0.707**± 0.042 |
| Delta-merge model on subset | original | 0.**799**± 0.075 | 0.729± 0.054 | 0.800± 0.071 | 0.833± 0.037 | 0.815± 0.042 | 0.833± 0.037 | 0.472± 0.100 |
| | SMOTE | 0.**850**± 0.056 | 0.738± 0.053 | 0.800± 0.071 | 0.715± 0.061 | 0.753± 0.049 | 0.715± 0.061 | **0.775**± 0.067 |

**Table S7.**

**Abbreviation of QVMFs**

| Component | Abbreviation | Full name | Short name |
|---|---|---|---|
| Artery | A | delta_**artery**_Mean Segment Tortuosity | Δ**A**mTort |
| Tortuosity | Tort | Delta_Mean Segment **Tortuosity** per Radius Bin 1 - 2 | Δm**Tort**_R12 |
| Vein | V | delta_**vein**_Mean Segment Length | Δ**V**mLen |
| Radius Bin 9 - 10 | _R910 | - | - |
| Radius Bin 1 - 2 | _R12 | - | - |
| Length | Len | delta_vein_Mean **Length** of Segments per Radius Bin 8 - 9 | ΔVm**Len**_R89 |
| Endpoint | End | Artery_**endpoint** | A**End** |
| delta | Δ | - | - |
| Segment | Seg | delta_Number of **Segments** per Radius Bin 9 - 10 | ΔN**Seg**_R910 |
| Number | N | delta_vein_**Number** of Segments per Radius Bin 2 - 3 | ΔV**N**Seg_R23 |
| Mean | m | delta_**Mean** Segment Length | Δ**m**Len |
| Partitioning | Part | vein_Segment **Partitioning** | VSeg**Part** |
| Line of Therapy | LOT | - | - |

- **File S1—Univariable and multivariable analyses of clinical and QVMFs associated with treatment response (.xlsx).**
- **File S2—paired t-test analysis of pre- and post-treatment QVMFs (.xlsx).**
